# Anions Govern Cell Volume: A Case Study of Relative Astrocytic and Neuronal Swelling in Spreading Depolarization

**DOI:** 10.1371/journal.pone.0147060

**Published:** 2016-03-14

**Authors:** Niklas Hübel, Ghanim Ullah

**Affiliations:** Department of Physics, University of South Florida, Tampa, FL 33620, United States of America; Albany Medical College, UNITED STATES

## Abstract

Cell volume changes are ubiquitous in normal and pathological activity of the brain. Nevertheless, we know little about the dynamics of cell and tissue swelling, and the differential changes in the volumes of neurons and glia during pathological states such as spreading depolarizations (SD) under ischemic and non–ischemic conditions, and epileptic seizures. By combining the Hodgkin–Huxley type spiking dynamics, dynamic ion concentrations, and simultaneous neuronal and astroglial volume changes into a comprehensive model, we elucidate why glial cells swell more than neurons in SD and the special case of anoxic depolarization (AD), and explore the relative contributions of the two cell types to tissue swelling. Our results demonstrate that anion channels, particularly Cl^−^, are intrinsically connected to cell swelling and blocking these currents prevents changes in cell volume. The model is based on a simple and physiologically realistic description. We introduce model extensions that are either derived purely from first physical principles of electroneutrality, osmosis, and conservation of particles, or by a phenomenological combination of these principles and known physiological facts. This work provides insights into numerous studies related to neuronal and glial volume changes in SD that otherwise seem contradictory, and is broadly applicable to swelling in other cell types and conditions.

## Introduction

Spreading depolarization (SD) is an abrupt process of prolonged cellular depolarization, which spreads through brain tissue in a wave–like manner. It is characterized by the breakdown of ion gradients, the depression of neuronal electrical activity, and an extreme shrinkage of extracellular space (ECS) resulting from the swelling of neurons and the surrounding glia cells [1, 2]. The cessation of electrical activity that goes along with SD is called spreading depression, but the terminology is ambiguous and many authors refer to the whole event as spreading depolarization instead. Strictly speaking the term anoxic depolarization (AD) refers to the special case of SD in which the neurons have inadequate blood supply, while SD is a more general term that also includes cell depolarizations caused by mechanical or electrical stimulation and by extracellular K^+^ elevation. Whenever we talk explicitly about both, AD and SD, the latter refers only to non–ischemic SDs as in migraine or in brain slice experiments with K^+^ elevation. Otherwise, SD means all kinds of spreading depolarizations. SD occurs frequently in patients with stroke and brain injury where it may cause progressive damage to the tissue at risk. Moreover, SD is believed to cause migraine [3–8], and there appears to be a correlation between brain susceptibilities to SD and epileptic seizures [2, 9–12].

The local processes during SD are understood as the interplay of neurons, astroglia cells (astrocytes), and the vascular system. The neuron releases large amounts of K^+^ into the ECS when it depolarizes. Astrocytes and blood vessels take up excess K^+^ and thereby help the neuron to repolarize and recover. The astrocytic K^+^ buffering ability is the result of inward–rectifying K^+^ channels, Na^+^ /K^+^ exchange pumps, cotransport processes, and spatial buffering. Besides K^+^, astrocytes take up large amounts of anions, mostly Cl^−^ [13–15]. In particular, a number of swelling–activated anion channels in glia cells and neurons are known [16–20].

The role of (astrocytic and neuronal) cell swelling during SD is of interest for several reasons. First, in brain slice experiments cell swelling changes the light transmittance index of the tissue, which makes SD visible to the experimentalist [21, 22]. More importantly, cell and tissue swelling can exacerbate SD and may lead to severe brain damage [19, 23]. In astrocytes, volume–activated anion channels may release large amounts of glutamate leading to excitotoxic damage [18]. Swelling of the brain as a whole may be harmful because it increases the intracranial pressure and can obstruct blood vessels [1, 24, 25].

The swelling mechanisms differ between neurons and astrocytes. Neurons lack functional aquaporins [26]. Although still debated, the K^+^ /Cl^−^ and Na^+^ /K^+^ /2Cl^−^ cotransporters are suspected to mediate the entry of water molecules into neurons [27, 28]. Astrocytes on the other hand, express aquaporins [29]. The clearance of excessive K^+^ due to high neuronal activity by astrocytes leads to osmotic gradients resulting in water influx through aquaporins and astrocytic dilation [30–32]. Regardless of the actual mechanism through which cell imports water, neurons and astrocytes share osmotic gradient as the common trigger for swelling.

In ischemia–induced AD and other SDs, a front of depolarization drains residual stored energy in compromised gray matter. During this electrophysiological event the ECS shrinks dramatically [21, 24, 25, 29, 33, 34]. The relative contribution of neuronal and astrocytic swelling to this shrinkage is a matter of debate. Some studies support the hypothesis that during these pathologies astrocytes swell more than neurons [24, 25, 29, 33, 35], while others claim the opposite [34]. We will comment on some of these studies in the discussion of our results.

Numerous single neuron models for investigating SD have been developed. The phenomenon is rather generic and is found in models with great physiological details [36–39] as well as in simplified Hodgkin–Huxley (HH) based descriptions of the neuron [40–43]. Also, the glia cell models vary in complexity, ranging from simple phenomenological modifications of the K^+^ dynamics [36, 40, 41, 43] to detailed membrane descriptions [4, 30]. With the help of these models, thresholds for SD ignition and recovery can be assessed. In particular, it can be analyzed how energy and oxygen supply, morphological parameters, and blood pressure affect the course of SD, how SD can be prevented, and when it is non–recoverable [6, 40, 41, 44–46].

Many computational studies do not include swelling dynamics at all [8, 36, 42, 43], and of those that do, most support the viewpoint that it does not drive SD. Swelling is only seen as a byproduct of the other processes and hence omitting it seems justifiable depending on the particular focus of a study. It should be noted though that one study claims the opposite by saying that SD cannot propagate without cell swelling [47]. What strikes is that most computational volume models only deal with neuronal swelling [40, 41, 47, 48]. An accurate model for glial volume dynamics during SD has not yet been developed despite the fact that astrocytes may swell dramatically. Ref. [37] has volume dynamics and a glial compartment, but the glia volume is unrealistically large (ten times the neuronal volume) and the focus is on the general amount of ECS shrinkage rather than the relative contribution from glial and neuronal swelling. The model in Ref. [30] deals with the astrocytic volume alone.

In this study, we develop a new comprehensive model that takes into account the dynamics of glial, neuronal, and ECS volumes simultaneously, and explains the quantative differences between glial and neuronal swelling during SD. We employ a standard HH–like description of the neuron and a phenomenological glia model for K^+^ buffering. The latter is extended to also include Cl^−^ uptake and Na^+^ release. This accounts for the glial anion channels, Cl^−^/K^+^–cotransport, and Na^+^/K^+^–exchange pumps [13, 14, 17]. This extension is not only physiologically reasonable, but also physically necessary to preserve electroneutrality.

Our new volume model is a refinement of a standard osmosis–based description, derived from first physical principles [22] and places a lower bound on the size of ECS. When this bound is reached due to neuronal and glial cell swelling, the volume of the whole tissue increases. Such boundary conditions and the cell swelling are usually implemented by an ad hoc volume model [37, 40, 41] that, however, is physically inconsistent as we show.

In our model, we are able to demonstrate that anion channels are intrinsically connected with cellular volume dynamics. This is experimentally confirmed [16, 20] and probably relates to the concept of volume–activated anion channels. With this understanding, we can explain why our model predicts the astroglia cells to swell more and remain swollen for longer than neurons. The reason is that astrocytic K^+^ buffering is electroneutral mainly because of Cl^−^ uptake. This implies that buffering goes along with a rather large net uptake of oppositely charged ions. In contrast, when neurons release K^+^ they take up similar amounts of the equally charged Na^+^ ions. Fluxes of Cl^−^ are much smaller. This implies much more pronounced glia swelling, which is consistent with several experimental studies on SD, AD, and stroke [24, 25, 29, 33].

In summary, our model is successful in explaining a number of experimental results on cellular volume dynamics during SD and AD. While the model contains phenomenological components, the quantitative differences between neuronal and glial swelling can be mainly understood from first physical principles. We only assume a glia cell that buffers K^+^ effectively and a neuron with HH–like membrane properties. All of our results are then implied by the principles of osmosis, electroneutrality, and an estimate of the glial anion channels, which also relates to electroneutrality. This is to our knowledge the first modeling attempt to understand the relative contribution of neuronal and glial swelling to ECS shrinkage in brain pathologies.

## Methods

### Ion and volume dynamics without glial buffering

#### Neuronal membrane

For our model, we employ a standard Hodgkin–Huxley (HH) formulation of the neuronal membrane [49]. It describes the evolution of the membrane potential *V* which is governed by the K^+^, Na^+^, and Cl^−^ ion currents *I*_*K*_, *I*_*Na*_, and *I*_*Cl*_ respectively. We also include a pump current *I*_*p*_ which is important for the ion dynamics. A capacitance *C*_*m*_ is assigned to the membrane. Similar models have been used in several other studies to model epileptic seizures, SD, and AD [41, 43, 44, 50–53].

The conductances of the ion channels depend on the gating variables *n* (K^+^ activation), *m* (Na^+^ inactivation), and *h* (Na^+^ activation), which correspond to opening probabilities of the respective gates. Their dynamics is given by the HH exponential functions *α*_*x*_ and *β*_*x*_ (for *x* ∈ {*n*, *m*, *h*}). The *m*–gate is extremely fast and we can use an adiabatic approximation for it. The full membrane model reads

$$\begin{array}{ccc} \frac{dV}{dt} & = & -\frac{1}{C_{m}}\left(I_{\text{Na}}+I_{K}+I_{\text{Cl}}+I_{p}\right)\;, \end{array}$$ (1)

$$\begin{array}{ccc} \frac{dn}{dt} & = & \phi \left(\alpha_{n}\left(1-n\right)-\beta_{n}n\right)\;, \end{array}$$ (2)

$$\begin{array}{ccc} \frac{dh}{dt} & = & \phi \left(\alpha_{h}\left(1-h\right)-\beta_{h}h\right)\;, \end{array}$$ (3)

and

$$\begin{array}{c} m=m_{\infty }=\frac{\alpha_{m}}{\alpha_{m}+\beta_{m}}\;. \end{array}$$ (4)

The timescale parameter *ϕ* is conventional. The voltage–dependent exponential functions are

$$\begin{array}{ccc} \alpha_{n} & = & \frac{0.01(V+34)}{1-\exp(-(V+34)/10)}\;, \end{array}$$ (5)

$$\begin{array}{ccc} \beta_{n} & = & 0.125\exp(-(V+44)/80)\;, \end{array}$$ (6)

$$\begin{array}{ccc} \alpha_{m} & = & \frac{0.1(V+30)}{1-\exp(-(V+30)/10)}\;, \end{array}$$ (7)

$$\begin{array}{ccc} \beta_{m} & = & 4\exp(-(V+55)/18)\;, \end{array}$$ (8)

$$\begin{array}{ccc} \alpha_{h} & = & 0.07\exp(-(V+44)/20)\;, \end{array}$$ (9)

$$\begin{array}{ccc} \beta_{h} & = & \frac{1}{1+\exp(-(V+14)/10)}\;. \end{array}$$ (10)

The currents *I*_*ion*_ (for *ion* ∈ {*K*, *Na*, *Cl*}) are all of the form

$$\begin{array}{c} I_{\text{ion}}=g_{\text{ion}}\left(V-E_{\text{ion}}\right)\;. \end{array}$$ (11)

Cl^−^ has a pure leak conductance $g_{\text{Cl}}=g_{\text{Cl}}^{l}$. The two gating–dependent conductances

$$\begin{array}{cc} g_{K} & =g_{K}^{l}+g_{K}^{g}n^{4}\;, \end{array}$$ (12)

$$\begin{array}{cc} g_{\text{Na}} & =g_{\text{Na}}^{l}+g_{\text{Na}}^{g}m^{3}h\;, \end{array}$$ (13)

are the sum of a leak conductance $g_{\text{ion}}^{l}$ and a gated term with a much higher maximal conductance $g_{\text{ion}}^{g}$. The Nernst potentials *E*_*ion*_ depend on the ion concentrations *ion*_*i*/*e*_ (for *ion*_*i*/*e*_ ∈ {*Na*_*i*/*e*_, *K*_*i*/*e*_, *Cl*_*i*/*e*_}) in the intra–/extracellular space ICS/ECS, and on the ion valence *z*_*ion*_:

$$\begin{array}{c} E_{ion}=\frac{26.64}{z_{ion}}\ln\left({\text{ion}}_{e}/{\text{ion}}_{i}\right) \end{array}$$ (14)

The coefficient 26.64 mV is computed from the ideal gas constant, the absolute temperature, and Faraday’s constant. All parameters are listed in Table 1. They are commonly used for this type of simplified single unit description [42, 43, 50–52], and the conductances and gating dynamics are based on an experimental estimation by Gutkin et al. [54].

**Table 1:** Model parameters.

Name	Value & unit	Description
*C*_*m*_	1 *μ*F/cm^2^	membrane capacitance
*ϕ*	3/msec	gating time scale parameter
$g_{Na}^{l}$	0.0175 mS/cm^2^	Na^+^ leak cond.
$g_{Na}^{g}$	100 mS/cm^2^	max. gated Na^+^ cond.
$g_{K}^{l}$	0.05 mS/cm^2^	K^+^ leak cond.
$g_{K}^{g}$	40 mS/cm^2^	max. gated K^+^ cond.
$g_{Cl}^{l}$	0.05 mS/cm^2^	Cl^−^ leak cond.
*ω*_*i*_	2,160 *μ*m^3^	ICS volume
*ω*_*e*_	720 *μ*m^3^	normal ECS volume
*F*	96,485 C/mol	Faraday’s constant
*A*_*m*_	922 *μ*m^2^	membrane surface area
*γ*	9.556e–2 $\frac{\text{fmol}}{\text{sec}}\frac{{\text{cm}}^{2}}{\mu \text{A}}$	conversion factor
*ρ*	6.8 *μ*A/cm^2^	max. pump current

#### Ion dynamics

**Fig 1:**
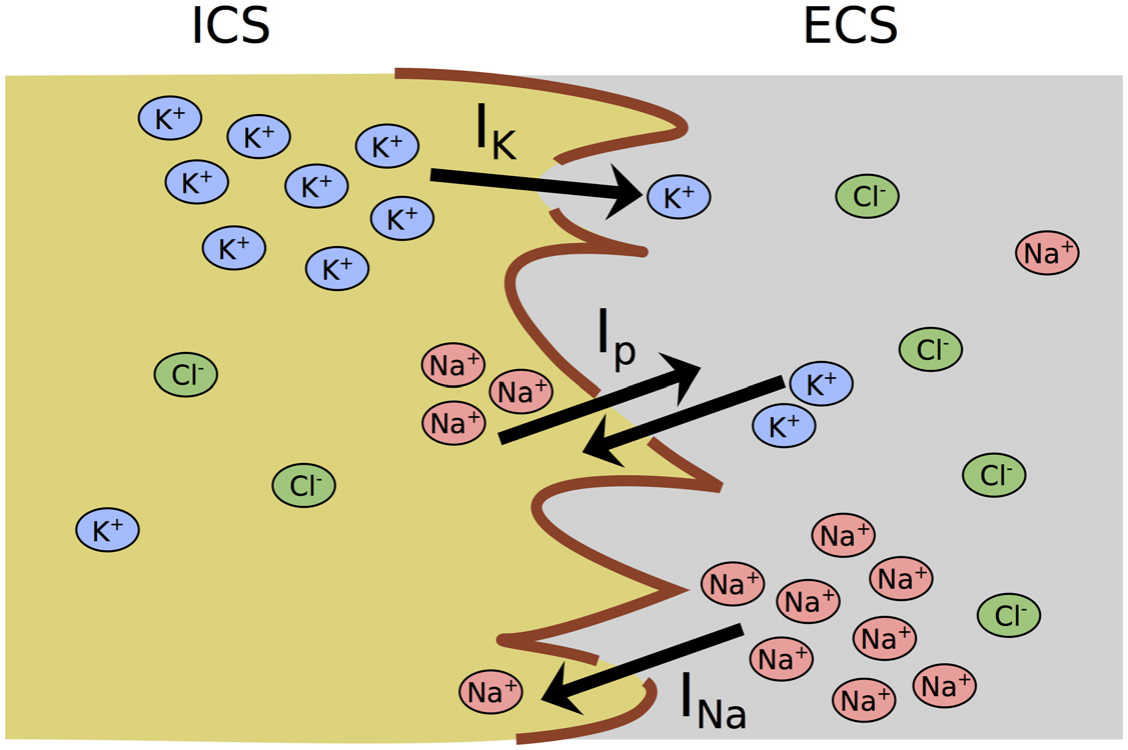
Model scheme for neuronal ion fluxes between the ICS and the ECS. The channel currents *I*_*K*_ and *I*_*Na*_ take K^+^ and Na^+^ across the membrane (red boundary line) from a region of high to a region of low concentration. The pump current *I*_*p*_ counteracts these fluxes and ion gradients are maintained. Under resting conditions Cl^−^ is in electrochemical equilibrium and *I*_*Cl*_ = 0 *μ*A/cm^2^ (not included in scheme).

The transmembrane currents *I*_*ion*_ go along with ion fluxes through the channels. The pumps exchange two extracellular K^+^ ions for three intracellular Na^+^ ions to keep the respective concentrations low. These processes are illustrated in Fig 1. To model the changes in the ion contents all currents must be converted to ion fluxes using the factor

$$\begin{array}{c} \gamma =\frac{A_{m}}{F}\;, \end{array}$$ (15)

which depends on Faraday’s constant *F* and the membrane surface area *A*_*m*_.

It is customary to model ion concentrations, but since we will also consider dynamical volume changes it is preferable to model the compartmental number of ions $N_{i/e}^{\text{ion}}$ instead. These only depend on currents, while concentrations are also affected by changes of the compartmental volumes *ω*_*i*/*e*_ and the rate equations are hence not as simple. For example,

$$\begin{array}{c} {\text{Na}}_{i}=\frac{N_{i}^{\text{Na}}}{\omega_{i}}\;\Rightarrow \;{\dot{\text{Na}}}_{i}=\frac{{\dot{N}}_{i}^{\text{Na}}}{\omega_{i}}-\frac{N_{i}^{\text{Na}}{\dot{\omega }}_{i}}{\omega_{i}^{2}}\;, \end{array}$$ (16)

where the dot indicates time derivative.

The simultaneous effect of channel currents and ion pumps leads to the following ion dynamics:

$$\begin{array}{ccc} \frac{\text{d}N_{i}^{\text{Na}}}{\text{d}t} & = & -\gamma (I_{{\text{Na}}^{+}}+3I_{p})\;, \end{array}$$ (17)

$$\begin{array}{ccc} \frac{\text{d}N_{i}^{K}}{\text{d}t} & = & -\gamma (I_{K^{+}}-2I_{p})\;, \end{array}$$ (18)

$$\begin{array}{ccc} \frac{\text{d}N_{i}^{\text{Cl}}}{\text{d}t} & = & \gamma I_{{\text{Cl}}^{-}}\;. \end{array}$$ (19)

The extracellular ion amounts follow from mass conservation, for example

$$\begin{array}{c} N_{e}^{\text{Na}}=N_{e}^{\text{Na},0}+N_{i}^{\text{Na},0}-N_{i}^{\text{Na}}\;, \end{array}$$ (20)

where superscript 0 denotes initial values.

Note that pumping is electrogenic and hence we have a net contribution of *I*_*p*_ to the rate Eq (1) for the membrane potential *V*. Pumping shall keep *Na*_*i*_ and *K*_*e*_ at low levels, and is therefore modeled to get stronger if these concentrations increase [51]:

$$\begin{array}{ccc} I_{p} & = & \rho {\left(1+\exp\left(\frac{25-{\text{Na}}_{i}}{3}\right)\right)}^{-1}\\ & & {\left(1+\exp\left(5.5-K_{e}\right)\right)}^{-1} \end{array}$$ (21)

Our simplified model is largely based on Refs. [50–52] and hence contains no active Cl^−^ transport via K^+^ − Cl^−^ cotransporter 2 (KCC2) and Na^+^ − K^+^ − Cl^−^ cotransporter 1 (NKCC1). The contribution of these processes can however be estimated from experimental data [41, 55]. For the depolarization scenarios that we consider below, the contribution of cotransporters turns out to be negligible in comparison to the Cl^−^ leak current. In experimental studies on the connection between Cl^−^ and volume dynamics, the Cl^−^ channels and the cotransporters can be blocked individually or simultaneously. We do not have this distinction in our model, but the cotransporter contribution overall is small.

The physiological resting state is characterized by large differences between the Nernst potentials, a membrane depolarization of about −70 mV, and huge intra– vs extracellular ion gradients. The values for our model are listed in Table 2. We will generally denote variable values at their initial resting conditions by a superscript zero, for example $K_{e}^{0}=4\;\text{mM}$. The Na^+^ and Cl^−^ concentrations differ slightly from standard values found in other models, because we do not employ any commonly used fixed leak currents. These depolarizing currents ensure the desired membrane depolarization, but are not physically reasonable, because they are not associated with ion fluxes. In fact, it can be shown that such currents change the mathematical structure of models for ion dynamics fundamentally and provide a false recovery mechanism in SD models [42, 43]. Instead our model has Cl^−^ fluxes which means that *I*_*Cl*_ cannot help membrane depolarization, since *V* = *E*_*Cl*_ under resting conditions. Hence we assume a slightly smaller reversal potential for Na^+^ to obtain the resting value of *V* from Table 2 and be otherwise consistent with the parameters from Refs. [50, 51, 56].

**Table 2:** Physiological resting conditions.

Name	Value & unit	Description
*V*	−67 mV	membrane potential
*n*	0.070	K^+^ activation
*m*	0.012	Na^+^ inactivation
*h*	0.978	Na^+^ activation
*Na*_*i*_	25.3 mM	conc. of Na^+^ in ICS
*Na*_*e*_	126.8 mM	conc. of Na^+^ in ECS
*K*_*i*_	128.6 mM	conc. of K^+^ in ICS
*K*_*e*_	4.0 mM	conc. of K^+^ in ECS
*Cl*_*i*_	10.1 mM	conc. of Cl^−^ in ICS
*Cl*_*e*_	124.7 mM	conc. of Cl^−^ in ECS
*X*_*i*_	147.2 mM	conc. of impermeants in ICS
*X*_*e*_	55.6 mM	conc. of impermeants in ECS
*E*_*Na*_	43 mV	Nernst potential Na^+^
*E*_*K*_	−92 mV	Nernst potential K^+^
*E*_*Cl*_	−67 mV	Nernst potential Cl^−^
*ω*_*i*_	2,160 *μ*m^3^	volume size of ICS
*ω*_*e*_	720 *μ*m^3^	volume size of ECS
$N_{i}^{\text{Na}}$	54.6 fmol	amount of Na^+^ in ICS
$N_{e}^{\text{Na}}$	91.3 fmol	amount of Na^+^ in ECS
$N_{i}^{K}$	277.7 fmol	amount of K^+^ in ICS
$N_{e}^{K}$	2.8 fmol	amount of K^+^ in ECS
$N_{i}^{\text{Cl}}$	21.7 fmol	amount of Cl^−^ in ICS
$N_{e}^{\text{Cl}}$	89.8 fmol	amount of Cl^−^ in ECS
$N_{i}^{X}$	318.0 fmol	amount of impermeants in ICS
$N_{e}^{X}$	40.0 fmol	amount of impermeants in ECS

The value of *ω*_*i*_ is a realistic soma volume [36], the membrane surface area is chosen such that the conversion factor *A*_*m*_/(*Fω*_*i*_) is consistent with previous models [50–52] and well within the range of surface area of the soma for pyramidal cells in the hippocampus [57]. When we deal with a glial compartment below we will assume that under resting conditions the glial and neural volume are approximately the same in the cortex [58]. The ECS volume size is then chosen to yield a whole tissue extracellular volume fraction of about 15% [59, 60]. The correctness of these initial volume ratios is important and it can, for example, be shown that a very large ECS leads to rather different dynamics [40, 46].

On the other hand the model dynamics are very robust with respect to cell geometries as reflected in different surface to volume ratios *A*_*m*_/*ω*_*i*_. With our choice of *A*_*m*_ we assume a nearly spherical cell shape. A cell model that includes the dendritic tree would have a larger surface to volume ratio *A*_*m*_/*ω*_*i*_ that is however still of a comparable order of magnitude (see Ref. [36]). Neural ion dynamics and SD in particular has been shown to arise from the interplay of distinct cellular processes that have hugely separated timescales: fast membrane dynamics (gating variables and membrane potential), slow transmembrane ion fluxes, and very slow glial and vascular ion regulation [43]. From this viewpoint a different cell geometry will only shift the timescale of transmembrane fluxes, which is inversely related to the surface to volume ratio, within the same order of magnitude. The general phase space structure as well as the expected dynamical behavior remain the same. In a more explicit analysis it has been shown that a fundamental bistability of reduced neuron models, that essentially governs the ion dynamics, is virtually independent of *A*_*m*_ within a range of two orders of magnitude [42].

#### Osmotic volume changes

During extreme events of ion dynamics such as AD or SD, neurons start to swell. The driving force behind these volume changes is an osmotic imbalance between the ECS and ICS. To quantify this imbalance, we look at the intra– and extracellular bulk concentrations

$$\begin{array}{c} \Pi_{i/e}={\text{Na}}_{i/e}+K_{i/e}+{\text{Cl}}_{i/e}+X_{i/e}\;. \end{array}$$ (22)

The condition for osmotic equilibrium is then

$$\begin{array}{c} \Pi_{i}=\Pi_{e}\;. \end{array}$$ (23)

Here we included some impermeant particles X. There must be additional matter to make sure that the initial state is in osmotic equilibrium and that the intra– and extracellular solutions carry no net charge. By the latter of these consistency conditions, the known concentrations of K^+^, Na^+^ and Cl^−^ from Table 2 imply anion concentrations of 143.8 mM and 6.1 mM in the ICS and ECS, respectively. These anion concentrations then imply that there must be at least 46.2 mM more impermeant (neutral) matter in the ECS such that this configuration is osmotically stable. We have chosen the amount of X in the ECS to be 40 fmol, which implies a slightly larger concentration (55.6 mM) than this minimum requirement. This means that the ICS also has some neutral particles in addition to ions. X is hence the sum of neutral particles and impermeant anions.

**Fig 2:**
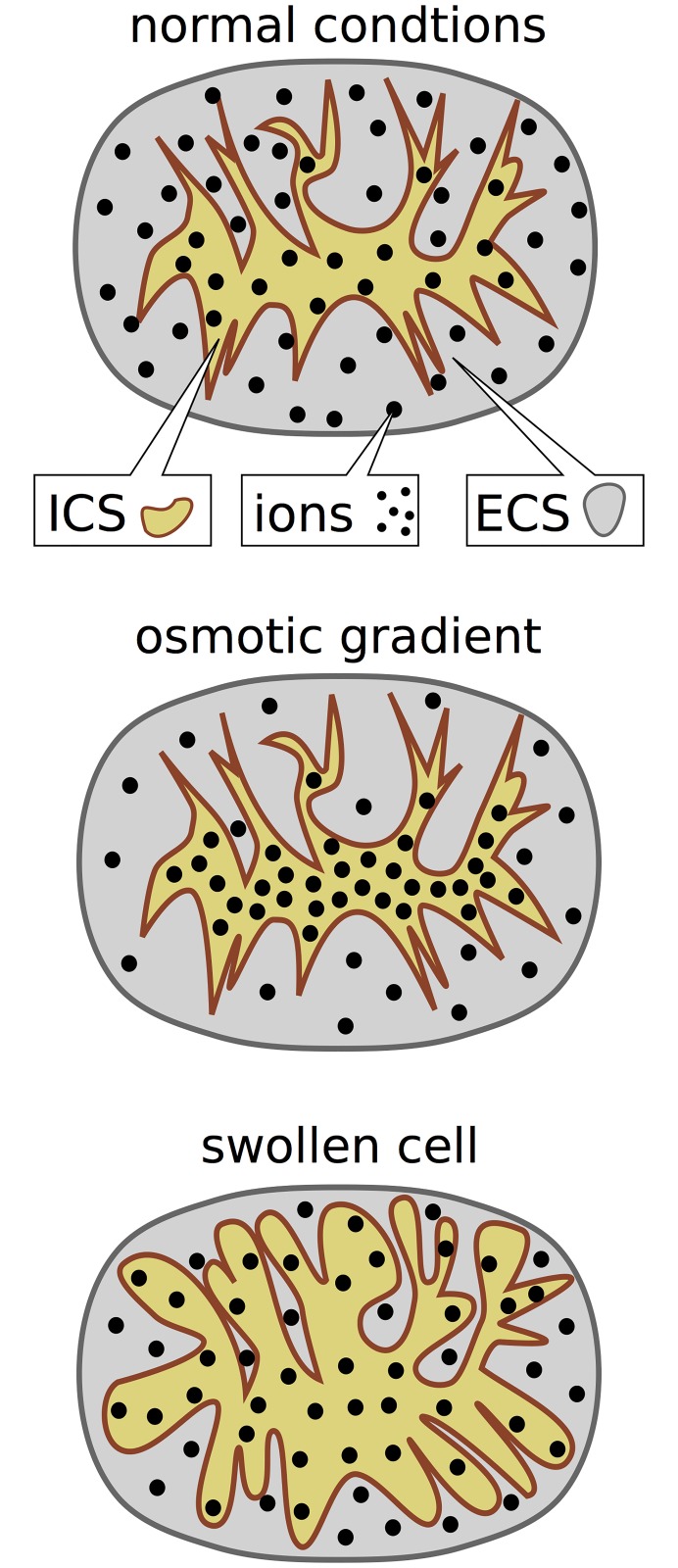
Illustration of osmotic cell swelling. Under normal conditions, the particle density in the ICS and ECS is equal. During SD and AD a net influx of ions into the cell leads to an osmotic gradient. The overall particle concentration in the ICS is higher. The cell swells in response to this imbalance until intra– and extracellular concentrations are equal again.

The breakdown of ion gradients during AD or SD goes along with a net flux of ions into the cell. This establishes an osmotic imbalance Π_*i*_ > Π_*e*_ and the cell swells to compensate for this. This general principle is illustrated in Fig 2. For now, we assume a constant total volume of the system

$$\begin{array}{c} \omega_{\text{tot}}=\omega_{i}+\omega_{e}\;. \end{array}$$ (24)

Computational models for SD often include volume dynamics by means of a phenomenological exponential ansatz [37, 39–41]. The expected intracellular equilibrium volume $\omega_{i}^{\text{eq}}$ based on the osmotic gradient is then given by

$$\begin{array}{c} \omega_{i}^{\text{eq}}=\omega_{i}^{0}\left(1.35-0.35\exp\left(\frac{\Pi_{e}-\Pi_{i}}{20}\right)\right)\;. \end{array}$$ (25)

The coefficients vary between models and are normally chosen to fit the range of experimentally observed cell swelling. It is however also possible to derive the expected volume directly from the equilibrium condition Eq (23) [22]. This derivation is best formulated in terms of the nonspecific particle amounts

$$\begin{array}{ccc} N_{i/e} & = & N_{i/e}^{\text{Na}}+N_{i/e}^{K}+N_{i/e}^{\text{Cl}}+N_{i/e}^{X}\;, \end{array}$$ (26)

$$\begin{array}{ccc} N_{\text{tot}} & = & N_{i}+N_{e}\;. \end{array}$$ (27)

For any given values of *N*_*i*_ and *N*_*e*_, the expected ICS volume $\omega_{i}^{\text{eq}}$ follows from the osmotic equilibrium condition

$$\begin{array}{ccc} \frac{N_{i}}{\omega_{i}^{\text{eq}}}=\frac{N_{e}}{\omega_{e}^{\text{eq}}}\;\Rightarrow \;\frac{N_{\text{tot}}}{\omega_{\text{tot}}} & = & \frac{N_{i}+N_{e}}{\omega_{i}^{\text{eq}}+\omega_{e}^{\text{eq}}}\\ & = & \frac{N_{i}+N_{i}\frac{\omega_{e}^{\text{eq}}}{\omega_{i}^{\text{eq}}}}{\omega_{i}^{\text{eq}}+\omega_{i}^{\text{eq}}\frac{\omega_{e}^{\text{eq}}}{\omega_{i}^{\text{eq}}}}\\ & = & \frac{N_{i}}{\omega_{i}^{\text{eq}}}\\ \Rightarrow \;\omega_{i}^{\text{eq}} & = & \omega_{\text{tot}}\;\frac{N_{i}}{N_{\text{tot}}}\;, \end{array}$$ (28)

where the first statement is equivalent to Eq (23). The derived relation is just the trivial statement that at equilibrium the particle concentration in each compartment is equal to the total particle concentration in the whole system.

The actual physiological mechanism that translates an osmotic imbalance into a volume change is an influx of water across the neuronal membrane which makes the cell swell. We do not model the details of this process, but instead employ a simple first–order process with a timescale *τ*_*ω*_ as in Refs. [37, 39–41]:

$$\begin{array}{c} \frac{\text{d}\omega_{i}}{\text{d}t}=\frac{\omega_{i}^{\text{eq}}-\omega_{i}}{\tau_{\omega }} \end{array}$$ (29)

The idea behind this ansatz is that, whatever the underlying mechanism may be, volume adjustments aim permanently towards the equilibrium. Lee and Kim describe this process explicitly by modeling the water flux caused by an osmotic gradient [22]. Unlike in Eq (29) their volume dynamics are driven by the difference between the inverses of the volumes. Our first order process is the linear approximation of their model, and we will argue below that it is a nearly perfect approximation. The reason is that within a reasonable range, the timescale *τ*_*ω*_ has hardly any effect on the volume dynamics and there is never a noticeable difference between *ω*_*i*_ and $\omega_{i}^{\text{eq}}$. This means that the physiological details of the volume adjustments can be neglected. A formal proof of this claim is given below. The extracellular volume follows by assuming that *ω*_*tot*_ is constant.

For the implementation of the model we have used the numerical integration software XPPAUT [61] that offers a range of solvers. We have compared our results for the classical Runge–Kutta method, the “stiff” and the “cvode” solver to eliminate numerical errors. The simulation code in .ode file format is made available from ModelDB [62] with accession number 187599. To run it you need the freely available XPPAUT software [63]. Alternatively the files can be opened with any text editor and the equations can be used to write code in another format.

## Results

### Donnan equilibrium: Why the exponential model violates the osmotic principle and why chloride is important

When the ion pumps are switched off (potentially due to oxygen–glucose deprivation) the system begins to evolve towards its thermodynamic equilibrium. Eventually all Nernst potentials are equal (see below), and during this transition from the normal resting state to thermodynamic equilibrium no charges are separated. It is noteworthy that the latter follows directly from the model Eqs (1) and (17)–(19). That is, the rate of change of the overall intracellular charge $N_{i}^{q}$ nearly vanishes:

$$\begin{array}{c} N_{i}^{q}:=N_{i}^{\text{Na}}+N_{i}^{K}-N_{i}^{\text{Cl}}\\ \;\Rightarrow \;{\dot{N}}_{i}^{q}=\gamma C_{m}\dot{V}=9.566\text{e}-5\frac{\text{fmol}}{\text{mV}}\;\dot{V}\\ \;\Rightarrow \;\Delta N_{i}^{q}=9.566\text{e}-5\frac{\text{fmol}}{\text{mV}}\;\Delta V\approx 0\\ \;\Rightarrow \;N_{i}^{q}\approx N_{i}^{\text{Na},0}+N_{i}^{K,0}-N_{i}^{\text{Cl},0} \end{array}$$ (30)

The second implication follows from integrating over time, and Δ symbolizes the difference of a quantity between the beginning and end of a chosen time window. The choice of times does not matter for our argument, because *V* will always range between −100 mV and 50 mV which makes the product of Δ*V* and *γC*_*m*_ extremely small for every time window. The amount of impermeant particles $N_{i}^{X}$ is constant and can hence be omitted in this consideration. The derived relation says that $N_{i}^{q}$ is practically constant. The same holds for the extracellular charge, which makes the final state with ceased pump activity a thermodynamic Donnan equilibrium. This symmetry has been pointed out before [42, 64, 65], but since electroneutrality will be crucial for volume dynamics, it is worthwhile to recall this derivation. Electroneutrality is not an assumption, but an inherent symmetry that relies on the different timescales of *V* and the ion concentrations [43]. Since astrocytes are cells with a similar membrane surface area and capacitance as neurons, they have a comparable numerical value for the factor *γC*_*m*_ and must therefore obey electroneutrality as well.

**Fig 3:**
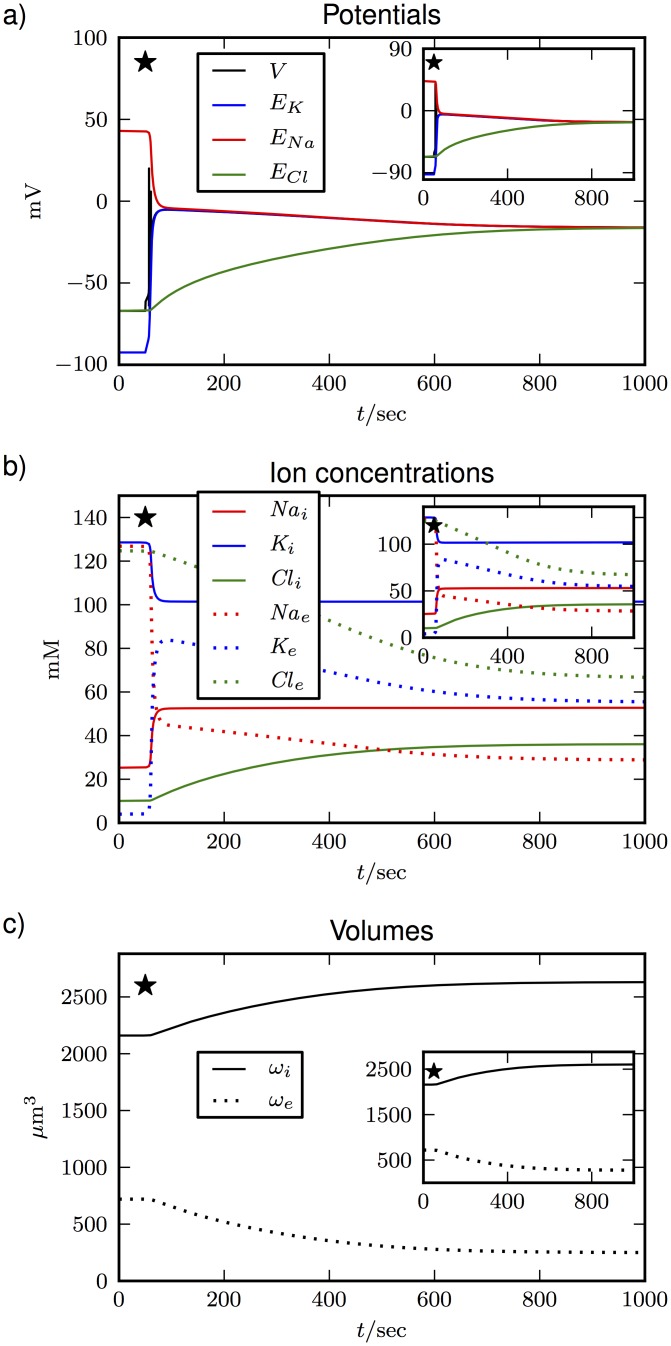
Transition from the physiological resting state to the Donnan equilibrium when the ion pumps are switched off after 50 sec (marked by the black star). The main plots show the evolution **(a)** of the membrane and Nernst potentials, **(b)** of the ion concentrations, and **(c)** of the volumes for the derived volume model based on Eq (28). The insets show the results for the exponential model from Eq (25).

During SD the system comes very close to the Donnan equilibrium, which makes this condition an important reference point of general interest. Furthermore modeling the transition of the cell from normal resting conditions to its thermodynamic equilibrium serves as a useful theoretical case study, which bears some general insights into volume dynamics. In the simulations shown in Fig 3, we compare the derived volume model based on Eq (28) (main plots) and the exponential model based on Eq (25) (insets). The plots show no apparent difference between the models with regard to the depolarization (Fig 3a), the breakdown of ion gradients (Fig 3b), and cell swelling (Fig 3c). However, a closer inspection of the final concentrations in Table 3 reveals that the exponential model is physically inconsistent, and we have Π_*i*_ > Π_*e*_ instead of osmotic equilibrium.

**Table 3:** Concentrations for the Donnan equilibrium in both volume models.

	derived model	exponential model
*Na*_*i*_	51.2 mM	51.4 mM
*K*_*i*_	98.4 mM	98.9 mM
*Cl*_*i*_	38.2 mM	37.8 mM
*X*_*i*_	81.6 mM	82.7 mM
Π_*i*_	269.4 mM	270.8 mM
*Na*_*e*_	35.5 mM	32.9 mM
*K*_*e*_	68.3 mM	63.3 mM
*Cl*_*e*_	55.0 mM	59.1 mM
*X*_*e*_	110.6 mM	83.8 mM
Π_*e*_	269.4 mM	239.1 mM

The exponential ansatz is often used in SD models, because the coefficients can be easily adjusted to reproduce the experimentally observed magnitude of cell swelling. However it violates the osmotic principle. We remark that this is not the result of our particular choice of coefficients. From Eq (25) we see that the equilibrium condition Π_*i*_ = Π_*e*_ corresponds only to one unique volume $\omega_{i}^{\text{eq}}$. So despite being motivated by osmosis, the exponential model yields an osmotic equilibrium for no other state than the initial condition. In the derived model there is no such constraint. In particular, also the Donnan equilibrium is osmotically balanced.

In the derived model there are no coefficients that we can choose freely to adjust the scope of cell swelling. The amount of impermeant matter X in the system influences volume dynamics and a large amount can limit the swelling magnitude. However, our assumed amounts of X are closed to the physically required minimum (see the above discussion), and we will instead introduce a model refinement to explicitly include a lower bound for the ECS volume when we introduce the glia model below.

**Fig 4:**
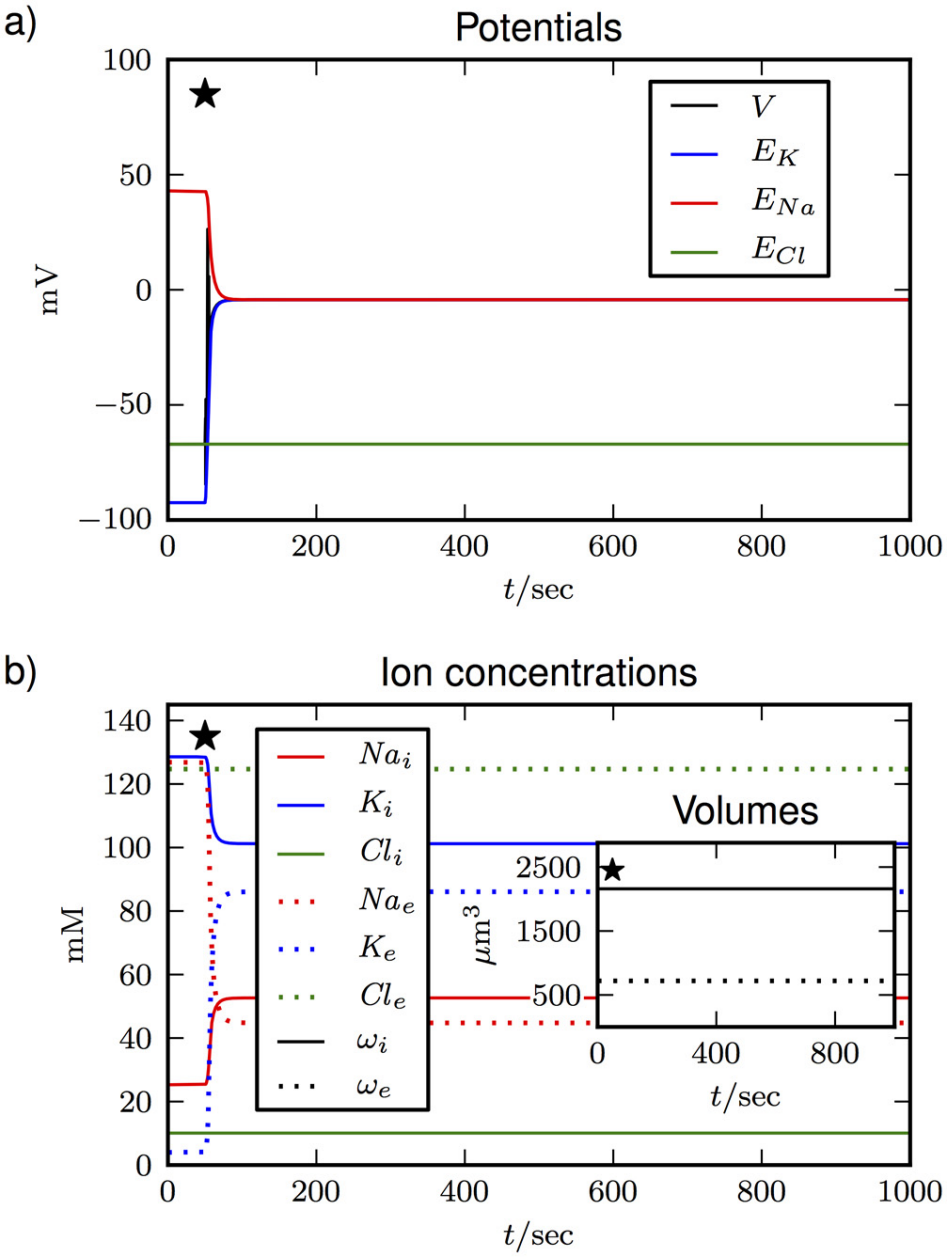
The same transition as in Fig 3 with blocked Cl^−^ channels. The pumps are switched off after 50 sec (marked by the star). The main plots show **(a)** potentials and **(b)** ion concentrations. Volumes are shown in the inset in **(b)**.

The simulations in Fig 3 show that volume dynamics is rather slow in comparison to Na^+^ and K^+^. Cl^−^ on the other hand evolves at a similar rate as the volume. Because of the similar timescales for the dynamics of volume and Cl^−^, we may conjecture that the dynamics are related. This is shown explicitly in Fig 4 where the Cl^−^ channels are blocked by setting $g_{\text{Cl}}^{l}$ to zero. Again, the system makes a transition to the Donnan equilibrium with reduced ion gradients and equal potentials, except for *E*_*Cl*_ which is constant. Only now this does not go along with any volume changes at all. This effect is gradual and swelling get slower the closer we get to *g_Cl_* = 0 mS/cm^2^.

There is a simple explanation for this. By virtue of electroneutrality $N_{i}^{q}$ is conserved. Since the Cl^−^ channels are blocked and $N_{i}^{q}$ is constant, also the sum of $N_{i}^{\text{Na}}$ and $N_{i}^{K}$ must be constant. Then also the overall sum *N*_*i*_ of intracellular particles is constant and according to Eq (28) the expected equilibrium volume $\omega_{i}^{\text{eq}}$ does not change. So because of electroneutrality, there can be no buildup of an osmotic imbalance without both, anion and cation fluxes. We can formalize this argument and derive the following expression for the equilibrium volume from Eqs (30) and (28):

$$\begin{array}{ccc} \omega_{i}^{\text{eq}} & = & \omega_{\text{tot}}\;\frac{N_{i}^{\text{Na}}+N_{i}^{K}+N_{i}^{\text{Cl}}+N_{i}^{X}}{N_{\text{tot}}}\\ & = & \omega_{\text{tot}}\;\frac{N_{i}^{\text{Na}}+N_{i}^{K}-N_{i}^{\text{Cl}}+2N_{i}^{\text{Cl}}+N_{i}^{X}}{N_{\text{tot}}}\\ & = & \omega_{\text{tot}}\;\frac{N_{i}^{q}+N_{i}^{X}+2N_{i}^{\text{Cl}}}{N_{\text{tot}}} \end{array}$$ (31)

The only nonconstant quantity in the last line of Eq (31) is $N_{i}^{\text{Cl}}$. Since osmosis and electroneutrality are fundamental principles, this connection between cellular volumes and anion fluxes also holds for the astroglia and other cell types with osmosis–driven volume dynamics. The role of anions in swelling processes has been pointed out in numerous experimental studies [13, 15–18]. For example, Ref. [16] shows in cultured astrocytes that changes in the cell cytoskeleton (which indicates changes of the shape) are sufficient and necessary to activate Cl^−^ channels. Figs 3 and 4 might be the first demonstration of such an intrinsic connection in a computational model.

With this new result we are now in a position to show that incorporating the specific biophysical details that underly cell swelling would have no impact on the behavior of our model. We have seen that cellular volumes respond to changes in Cl^−^. The dynamics of Cl^−^ are much slower than those of the other ions, because the channel only has a small leak conductance of 0.05 mS/cm^2^. This corresponds to a permeability of about 0.05 *μ*m/sec. The timescale of Cl^−^ is inversely related to this permeability and is about 25 sec [43]. Volume changes are mediated by fluxes of water across the neuronal membrane. For cells exhibiting aquaporins, the water permeability is of the order 1 m/sec which is more than seven orders of magnitude larger and would yield a timescale of about 0.0025 msec [22]. The value we have chosen is 50 msec instead and corresponds to a much (20,000 times) lower water permeability, but even then volume dynamics are extremely fast compared to Cl^−^. We have tested different timescales and the results from Fig 3 are virtually unchanged for any choice of *τ*_*ω*_ between values as large as 1 sec and adiabatic volume dynamics with

$$\begin{array}{c} \tau_{\omega }=0\;\text{and}\;\omega_{i}\equiv \omega_{i}^{\text{eq}}\;. \end{array}$$ (32)

This discussion of timescales shows that the driving force behind volume dynamics is extremely slow, and in comparison to that the transient volume adjustments of the cell are practically instantaneous. There is never a noticeable difference between *ω*_*i*_ and $\omega_{i}^{\text{eq}}$, even if we use a timescale that is many orders of magnitude larger than what would be implied by aquaporins. Hence we can perfectly employ the adiabatic approximation Eq (32). This implies that the osmotic equilibrium condition

$$\begin{array}{c} \frac{N_{i}}{\omega_{i}}=\frac{N_{e}}{\omega_{e}}=\frac{N_{\text{tot}}}{\omega_{\text{tot}}} \end{array}$$ (33)

is satisfied at all times. While this argument applies to our neuron model, the large water permeability of the astrocyte membrane obviously implies that glial volume adjustments will also be practically instantaneous.

We remark that finding the same volume dynamics for very fast and very slow timescales implies that a more detailed volume model will behave exactly the same way. This can be formally proven as follows. Let us denote the solution of a correct biophysical volume model by $\omega_{i}^{*}$ and let the change rate be given by a function $f(\omega_{i}^{\text{eq}},\omega_{i}^{*})$. Then we can rearrange the rate equation

$$\begin{array}{c} \frac{d\omega_{i}^{*}}{dt}=f\left(\omega_{i}^{\text{eq}},\omega_{i}^{*}\right)=\frac{\omega_{i}^{\text{eq}}-\omega_{i}^{*}}{\tau (\omega_{i}^{\text{eq}},\omega_{i}^{*})} \end{array}$$ (34)

and extract a positive timescale function $\tau (\omega_{i}^{\text{eq}},\omega_{i}^{*})$. This factorization is always possible if we assume the volume to aim permanently towards the equilibrium. The timescale function contains all biophysical details and may depend on other quantities besides $\omega_{i}^{*}$ and $\omega_{i}^{\text{eq}}$. We have mentioned above that the first oder process of Eq (29) yields the same solutions for extremely small and extremely large timescales. We will now use this fact to derive an expression for $\omega_{i}^{*}$. Let *τ*_1_ and *τ*_2_ be very large and very small, say 1 sec and 1e–15 sec, respectively. We denote the corresponding first order solutions by $\omega_{i}^{\left(1\right)}$ and $\omega_{i}^{\left(2\right)}$, and we have checked (not shown) that they are the virtually identical.

Regardless of the details of the biophysical model, we can always expect its timescale to lie somewhere between these extremes:

$$\begin{array}{c} \tau_{2}\leq \tau \left(\omega_{i}^{\text{eq}},\omega_{i}^{*}\right)\leq \tau_{1} \end{array}$$ (35)

For a swelling event ($\omega_{i}^{\text{eq}}\geq \omega_{i}^{*},\omega_{i}^{\left(1\right)},\omega_{i}^{\left(2\right)}$) this implies the following upper and lower bound on the swelling rate of the biophysical model:

$$\begin{array}{c} \frac{d\omega_{i}^{\left(1\right)}}{dt}\leq \frac{d\omega_{i}^{*}}{dt}\leq \frac{d\omega_{i}^{\left(2\right)}}{dt} \end{array}$$ (36)

Integrating this equation yields

$$\begin{array}{c} \omega_{i}^{\left(1\right)}\leq \omega_{i}^{*}\leq \omega_{i}^{\left(2\right)}\;, \end{array}$$ (37)

and since $\omega_{i}^{\left(1\right)}$ and $\omega_{i}^{\left(2\right)}$ are equal we have

$$\begin{array}{c} \omega_{i}^{*}=\omega_{i}^{\left(1\right)}=\omega_{i}^{\left(2\right)}\;. \end{array}$$ (38)

This obviously implies that $\omega_{i}^{*}$ can also be approximated adiabatically. Please note that this proof relies solely on the fact that Cl^−^ fluxes are very slow. It is easily generalized to cell shrinkage or a sequence of swelling and shrinkage.

### Free energy–starvation and osmosis

There is a fully developed phase space analysis of SD dynamics that links the typical course of SD events—breakdown of ion gradients, prolonged depolarization and sudden repolarization—to a metastable condition called ‘free energy–starvation’ (FES) [43]. This viewpoint is also consistent with experimental data [66]. FES is reminiscent of the Donnan equilibrium in terms of membrane depolarization, closeness of Nernst potentials, reduction of ion gradients and the cell being dysfunctional. For models where ion homeostasis relies on the Na^+^/K^+^–exchange pumps alone FES is stable and coexists with the normal resting state despite normal pump activity. In combined neuron–glia models FES is metastable and after a strong enough stimulation the cell will be ‘free energy–starved’ for about 80 sec. The combined effort of the ion pumps and other mechanisms of ion regulation slowly destabilizes FES and eventually results in a sharp characteristic repolarization drop that is common to all SD models (see below and Refs. [6, 36, 40–43]).

FES has not been shown in a model with volume dynamics and we will now demonstrate the existence. This is crucial, because it implies that our general understanding of the distinct roles of ion pumps and glia cells in ion homeostasis, and more specifically our interpretation of SD as a process of transitions between FES and the normal resting state remains valid. While we will not apply the phase space analysis making this connection explicit in this article, it is reassuring that the quantitative methods for the derivation of thresholds involved in SD remain applicable [43, 46]. When the role of FES in SD is appreciated, it is possible to model the depolarization process as an isolated event, namely as the transition from normal conditions to FES, in a model without glia (see discussion for an example).

**Fig 5:**
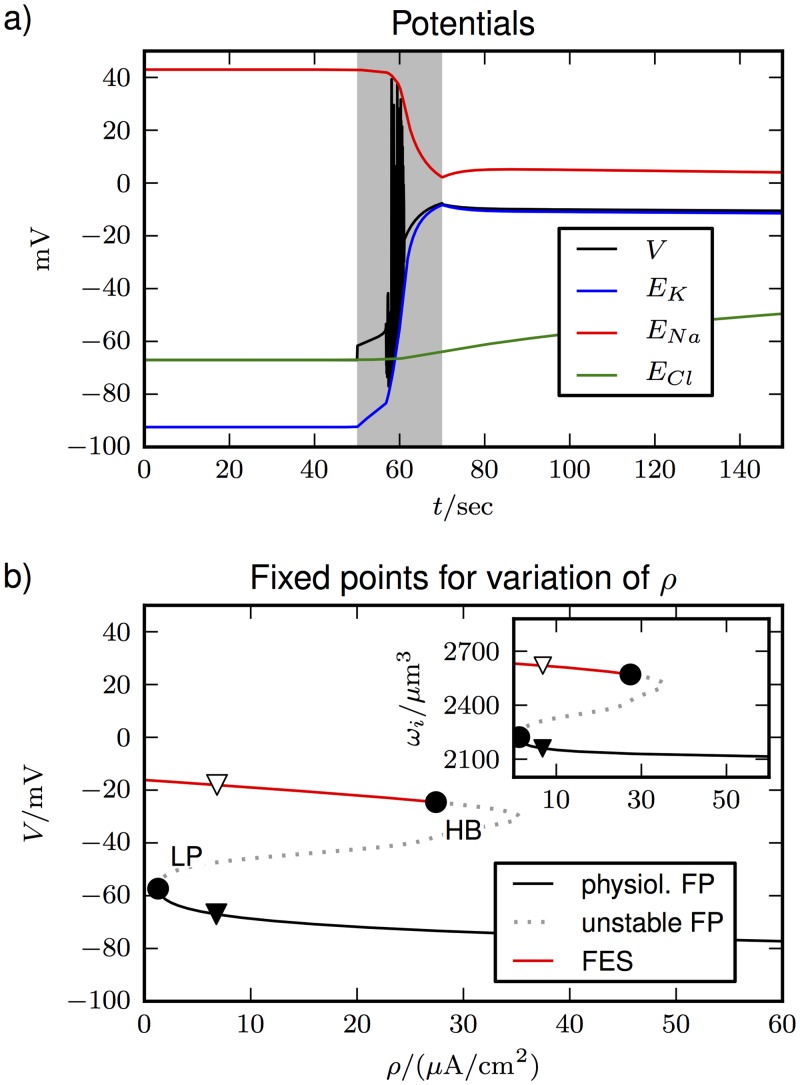
**(a)** Transition from physiological conditions to FES because of interrupted pump activity (shaded region). The cell depolarizes and Nernst potentials get close to each other. **(b)** The fixed point (FP) diagram shows that a polarized physiological fixed point and depolarized FES coexist for a large range of *ρ*–values. FES ends in a Hopf bifurcation (HB) at a higher pump rate. The physiological branch becomes unstable in a limit point bifurcation (LP) at a low pump rate. The inset shows the ICS volume, which is larger for FES (swollen cell). The initial and asymptotic states for long times from **(a)** are marked by a black and white triangle, respectively.

In Fig 5a we show the existence of FES by a time series in which the pump activity is interrupted for 20 sec (shaded region). The cell depolarizes in a very similar manner as in Fig 3a. The re–activated pumps do not recover the cell’s polarization and potential differences, but instead a new asymptotic state with all potentials near 0 mV is attained. These dynamics correspond to the bistable fixed point structure shown in Fig 5b. To unveil this bistability of states, the pump rate *ρ* was varied within a certain range. The system has a stable physiological fixed point branch (solid black line) on which also the resting state from Table 2 lies (black triangle at *ρ* = 6.8 *μ*A/cm^2^). FES lies at strongly depolarized values and is stable (solid red line) up to high pump rates. The asymptotic state of the time series in Fig 5a is marked by the white triangle. The inset shows that FES goes along with marked cell swelling, which demonstrates that this condition shares the whole symptomatology of the depolarized state during SD.

### Electroneutral buffering and swelling of astrocytes

#### Electroneutral glia model

Local SD dynamics are a sequence of events. First, the neuron depolarizes and goes into FES. In FES astroglial buffering becomes effective and after about 80 sec the combined effort of buffering and ion pumps recovers the neuron. Without the astrocytes FES would be permanent.

To understand the role of astroglia, it is important to note that SD is most prominently characterized by an extreme elevation of the extracellular K^+^ concentration. The astrocytes take up the excess K^+^ ions and thereby help the neuron to recover. Glia cells are complex systems and amongst other processes, inward rectifying K^+^ currents, spatial buffering, and cotransporters contribute to the K^+^ uptake [13, 14, 17]. We do not attempt to model such details, but instead assume the presence of a functional glia cell with given buffering properties. This is described by a phenomenological equation for the K^+^ uptake rate, which increases for high values of *K*_*e*_ [51]:

$$\begin{array}{c} \lambda^{\text{upt}.}=\lambda_{1}(1.0+\exp{\left(\frac{5.5-K_{e}}{2.5}\right))}^{-1} \end{array}$$ (39)

We have to assume a constant K^+^ release rate λ^*rel.*^ so that under physiological resting conditions no ions leak into the glia cell. The K^+^ flux into the glial cell is then

$$\begin{array}{c} J_{\text{glia}}=\lambda^{\text{upt}.}-\lambda^{\text{rel}.}\;. \end{array}$$ (40)

We incorporate buffering into our model by introducing the variable Δ*N*^*K*^ which measures the amount of K^+^ that has gone from the ECS into the glia cell:

$$\begin{array}{ccc} N_{e}^{K} & = & N_{e}^{K,0}+N_{i}^{K,0}-N_{i}^{K}-\Delta N^{K}\;, \end{array}$$ (41)

$$\begin{array}{ccc} \frac{\text{d}\Delta N^{K}}{\text{d}t} & = & J_{\text{glia}}\;. \end{array}$$ (42)

This is a common description of glial buffering [8, 36, 41, 43], but it violates electroneutrality. The glia cell removes positive charges from the ECS, while it does not replace them with other positive ions or remove negative charges as well. For models without volume dynamics this is negligible, but we have seen in Sec. “Donnan equilibrium …” how important electroneutrality becomes when osmosis is included. Hence we propose the following extension of the glia model:

$$\begin{array}{ccc} N_{e}^{\text{Na}} & = & N_{e}^{\text{Na},0}+N_{i}^{\text{Na},0}-N_{i}^{\text{Na}}-\Delta N^{\text{Na}}\;, \end{array}$$ (43)

$$\begin{array}{ccc} N_{e}^{\text{Cl}} & = & N_{e}^{\text{Cl},0}+N_{i}^{\text{Cl},0}-N_{i}^{\text{Cl}}-\Delta N^{\text{Cl}}\;, \end{array}$$ (44)

$$\begin{array}{ccc} \Delta N^{\text{Na}} & = & \left(\chi -1\right)\Delta N^{K}\;, \end{array}$$ (45)

$$\begin{array}{ccc} \Delta N^{\text{Cl}} & = & \chi \Delta N^{K}\;. \end{array}$$ (46)

We have introduced the parameter *χ* ∈ [0, 1] to choose a combination of Na^+^ release (*χ* = 0) and Cl^−^ uptake (*χ* = 1) that goes along with the primary K^+^ buffering process. Our choice is *χ* = 0.8, because there is abundant experimental evidence of Cl^−^ channels being crucially involved in astrocytic volume dynamics during SD [1, 13, 14, 17, 18], while Na^+^ fluxes seem to be much smaller [1, 35] and Na^+^ channels may even not exist on some glia cells [13]. To our knowledge the magnitude of Na^+^ and Cl^−^ fluxes has not been compared in experiments and our choice is only a rough estimate. In computational models of glia cells electroneutrality is sometimes violated and no major anions are described at all [4, 67]. Models that respect electroneutrality do typically not evaluate explicitly how large the Na^+^ and Cl^−^ fluxes are, but the models are set up such that K^+^ fluxes dominate over Na^+^ [30, 37]. All these studies support our choice of a rather large value for *χ*. Below we will show that choosing *χ* too small, i.e., assuming large Na^+^ fluxes would in fact prevent recovery from SD and hence severely impair glial ion regulation (see Sec. “Cell swelling during spreading depolarization”).

With our new description of electroneutral buffering, it is straightforward to extend the cell swelling model to the glial compartment. The overall amount *N*_*g*_ of particles inside the glia cell changes according to the above described uptake and release processes:

$$\begin{array}{c} N_{g}=N_{g}^{0}+\Delta N^{K}+\Delta N^{\text{Na}}+\Delta N^{\text{Cl}} \end{array}$$ (47)

The initial glia volume $\omega_{g}^{0}$ is set to be equal to the initial volume of the neuron $\omega_{i}^{0}$ [58]. Then for the initial state to be in osmotic equilibrium the amount of particles in the neuron and glia cell must be equal:

$$\begin{array}{c} N_{i}^{0}=N_{g}^{0} \end{array}$$ (48)

Note that it is not necessary to specify content of the particular types of particles in the glia cell.

In this extended model the total amount of particles and the total volume of the system are

$$\begin{array}{ccc} N_{\text{tot}} & = & N_{i}+N_{e}+N_{g}\;, \end{array}$$ (49)

$$\begin{array}{ccc} \omega_{\text{tot}} & = & \omega_{i}+\omega_{e}+\omega_{g}\;. \end{array}$$ (50)

We assume that *N*_*tot*_ is constant, but we refine the volume model such that swelling of the neuron and the glia cell may lead to whole tissue swelling when the ECS becomes too small. For very low values of *ω*_*e*_ further shrinkage is inhibited and instead *ω*_*tot*_ increases. This new volume model is introduced below.

The derivation of the osmotic equilibrium volume $\omega_{i}^{\text{eq}}$ in Eq (28) remains valid. Using the adiabatic approximation we have

$$\begin{array}{ccc} \omega_{i/e/g} & = & \omega_{i/e/g}^{\text{eq}}\;,\\ \omega_{i/e/g} & = & \omega_{\text{tot}}\;\frac{N_{i/e/g}}{N_{\text{tot}}}\;. \end{array}$$ (51)

Note that for another initial glia volume $\omega_{g}^{0}$ (and another matter amount $N_{g}^{0}$ that is consistent with an initial osmotic equilibrium) our model will produce the same absolute volume changes. The fraction *ω*_*tot*_/*N*_*tot*_ is independent of our choice and accordingly the volume change Δ*ω*_*g*_ that is induced by a change in the glial matter content Δ*N*_*g*_ will be the same. So the absolute contribution of the glia cell to ECS shrinkage and tissue swelling (see below) is unaffected by the initial assumption.

If the system size is constant the extracellular volume decreases linearly with *N*_*e*_:

$$\begin{array}{c} \omega_{\text{tot}}=\omega_{\text{tot}}^{0}\;\Rightarrow \;\omega_{e}=\omega_{\text{tot}}^{0}\;\frac{N_{e}}{N_{\text{tot}}} \end{array}$$ (52)

This formula assumes no lower bound for the *ω*_*e*_ and it could theoretically become arbitrarily small. However, in a real system the shrinkage of the ECS is limited by the branched cell structures and the folded membranes. Neurons and astrocytes cannot fill out the ECS entirely.

To account for this, we assume that Eq (52) does not hold for very small *N*_*e*_–values. Instead shrinkage slows down and the ECS remains larger than it would be for a constant system size. When this happens the particle density in the ECS decreases, and so does the particle density in the entire system:

$$\begin{array}{c} \omega_{e}>\omega_{\text{tot}}^{0}\;\frac{N_{e}}{N_{\text{tot}}}\;\Rightarrow \;\frac{N_{\text{tot}}}{\omega_{\text{tot}}^{0}}>\frac{N_{e}}{\omega_{e}}=\frac{N_{\text{tot}}}{\omega_{\text{tot}}} \end{array}$$ (53)

This implies a larger system size $\omega_{\text{tot}}>\omega_{\text{tot}}^{0}$. Instead of a further shrinking ECS, we now have swelling of the whole tissue.

To model a lower bound of the ECS volume, we use a fitted function for *ω*_*e*_ that gradually deviates from Eq (52) and does not become smaller than 140 *μ*m^3^:

$$\begin{array}{c} \omega_{e}=210+\frac{0.93N_{e}\frac{\omega_{\text{tot}}^{0}}{N_{\text{tot}}}-111}{1+\exp\left(0.005\left(105-N_{e}\frac{\omega_{\text{tot}}^{0}}{N_{\text{tot}}}\right)\right)} \end{array}$$ (54)

**Fig 6:**
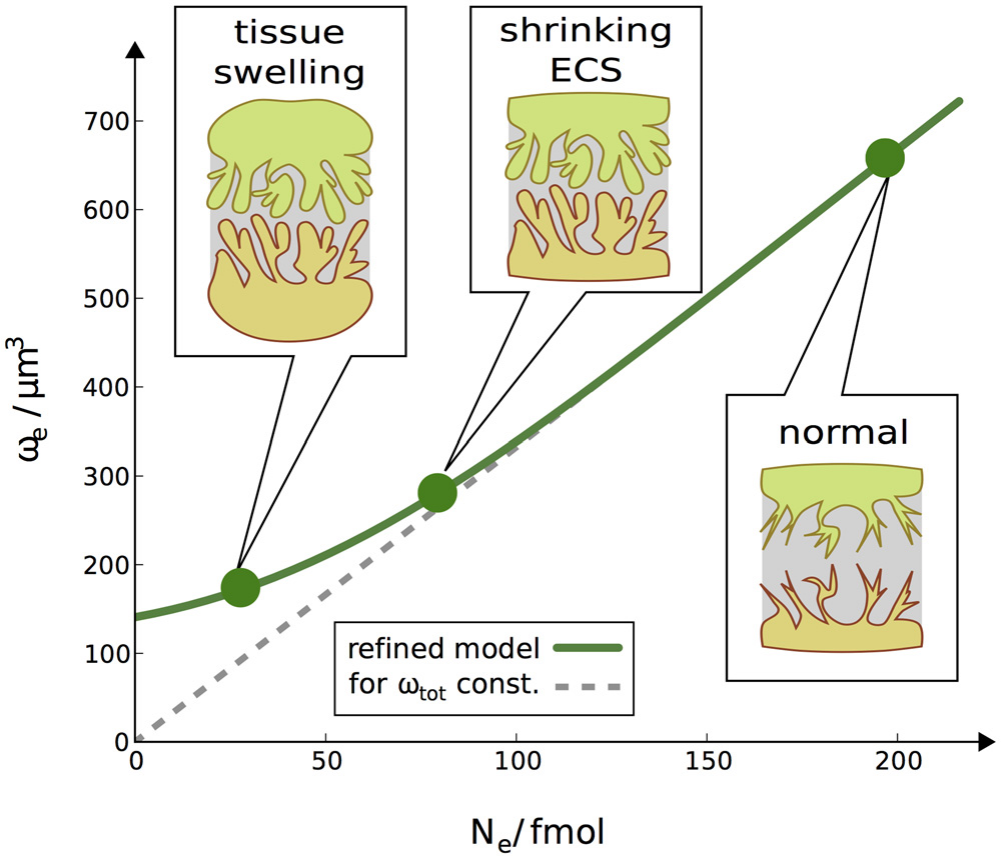
Illustration of the refined volume model for a system consisting of a neuron (orange cell in drawings), a glia cell (green cell), and the ECS between them (shaded region). The dashed grey curve shows unbounded shrinkage of the ECS for a fixed system size. The green curve shows our refined model with a lower bound of the ECS. It gradually deviates from the unbounded model. Instead of further shrinkage of the ECS there is tissue swelling for very high particle contents inside the cells (low values of *N*_*e*_).

This volume model is illustrated in Fig 6. The glia model is compatible with the previous resting state in Table 2. The resting state values of the additional glia variables and all new model parameters are listed in Table 4.

**Table 4:** Resting values and parameters for the glia model.

Name	Value & unit	Description
*χ*	0.8	Cl^−^ uptake factor
λ_1_	1.75 fmol/sec	K^+^ buffering rate constant
λ^*rel.*^	6.2e–1 fmol/sec	K^+^ release rate
*N*_*g*_	672 fmol	particle amount in glia
Δ*N*^*K*^	0 fmol	change of K^+^ amount in glia
Δ*N*^*Na*^	0 fmol	change of Na^+^ amount in glia
Δ*N*^*Cl*^	0 fmol	change of Cl^−^ amount in glia
*ω*_*g*_	2,160 *μ*m^3^	glia volume

#### Cell swelling during spreading depolarization

With the inclusion of the glial compartment, an interruption of pump activity leads to typical SD dynamics. Along with the pumps, glial ion regulation is also interrupted. Both processes are dependent on oxygen and glucose supply and are likely to be simultaneously affected in living tissue as well as in brain slice experiments.

**Fig 7:**
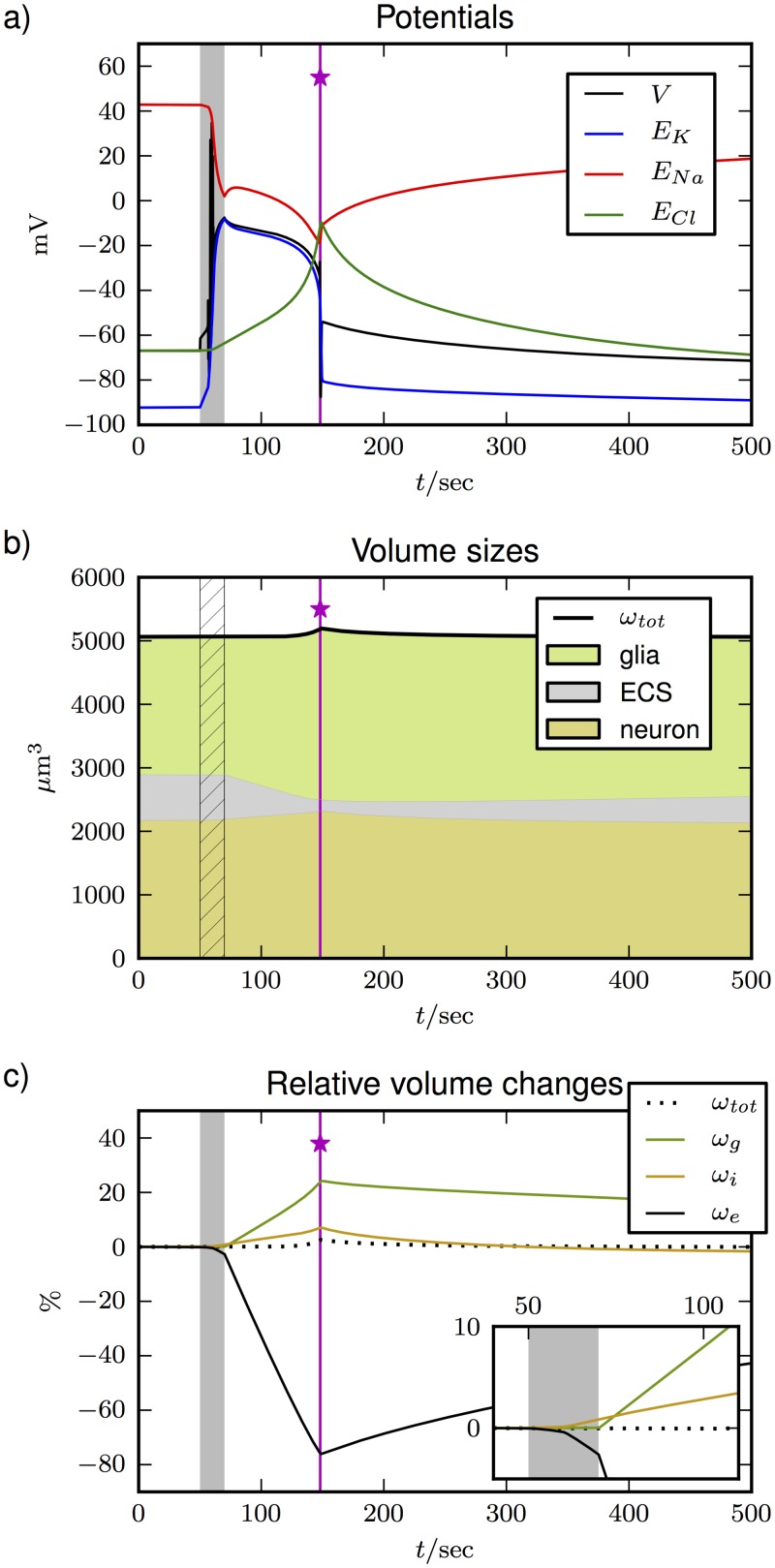
SD dynamics in model system consisting of a neuron, glial compartment, and the ECS. SD is initiated by interrupting pump activity and glial ion regulation for 20 sec (shaded or hatched region). In **(a)** the evolution of potentials is shown. During pump interruption the cell depolarizes and differences between *V*, *E*_*K*_ and *E*_*Na*_ become small. The system repolarizes abruptly and potential differences are rebuilt after about 150 sec (repolarization point indicated by vertical pink line and a star). The volume of the whole system and the respective portion of the neuron, ECS, and the glia cell are shown in **(b)**. Relative changes of the three compartments and the full system are shown in **(c)** with an inset for finer resolution between 40 sec and 110 sec.

**Fig 8:**
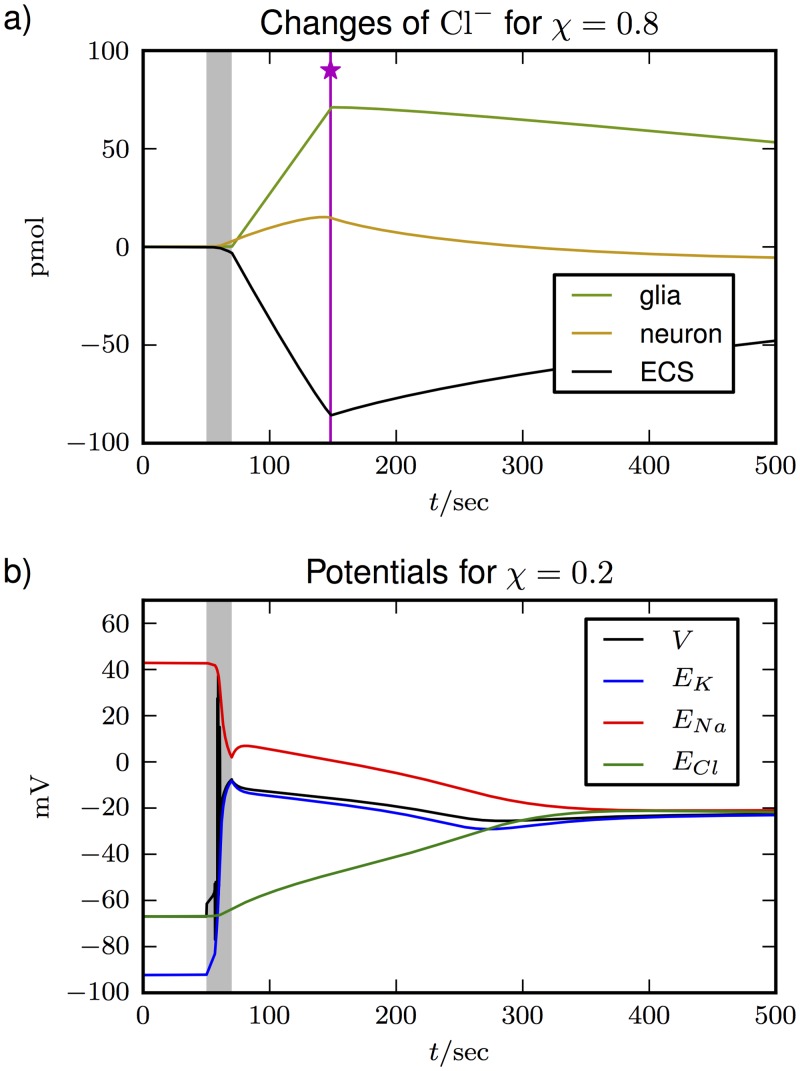
Cl^−^ fluxes and uptake factor *χ*. In **(a)**, changes in the Cl^−^ content in the three compartments during SD with *χ* = 0.8 (as in Fig 7) are shown. Pump interruption and the recovery point are indicated as before. In **(b)**, we used *χ* = 0.2 which means less Cl^−^ uptake and more Na^+^ release. The cell does not recover from depolarization, but remains in FES.

Fig 7a shows the familiar course of events. The pump interruption (shaded region) causes a burst of spikes resulting in membrane depolarization. The depolarization is sustained for about 80 sec, after which the cell repolarizes (at the vertical pink line marked with a star) and recovers slowly but fully. This is the expected effect of glial buffering and our electroneutral extension of the model by Eqs (43)–(46) does not alter the course of events. Note, however, that our choice of *χ* = 0.8 is crucial and in Fig 8b we see that recovery fails for too small values of *χ* (less Cl^−^ uptake and more Na^+^ release by the glia cell). So, too much release of Na^+^ interferes with recovery and the uptake of anions is essential.

Fig 7b shows the evolution of volumes. The total volume of the system is presented by the top black curve. The colored regions indicate the portion taken by ECS (grey), the neuron (orange), and the glial compartment (green). Relative changes are shown in Fig 7c with an inset for a finer resolution between 40 and 110 sec. Pump interruption is indicated by a shaded or hatched region and the repolarization point is marked by the vertical pink line (with a star) in each panel.

During pump interruption the neuron depolarizes almost completely, but it only grows marginally into the ECS (see inset). The glia cell does not exchange particles with the ECS during this initiating period and hence maintains its size. When pumps and glia are again functional after 70 sec the ECS begins to shrink much faster. This rapid shrinkage goes on until the repolarization point is reached (Fig 7b and 7c) and recovery sets in. During ECS shrinkage, the glial component grows significantly larger than the neuron, which can be clearly seen from the relative changes shown in Fig 7c. At the repolarization point, the glia cell has grown by 24%, while neuronal swelling with a maximum of 4% is less pronounced. After about 120 sec, the growth of the cellular volumes leads to tissue swelling. The whole system’s size increases by 2.6% until the repolarization point is reached and all volumes slowly recover. During the entire SD event the ECS shrinks by more than 75%. The amount of ECS shrinkage in our model is consistent with experiments reporting a reduction by 70% [1, 68]. Specific percentages for the amount of neural and glial swelling in experimental studies are hardly available. Neural swelling (most notably dendritic beading) and glia swelling are often made visible through imaging techniques, but not further quantified [21, 69]. Still Risher et al. [24] and Zhou et al. [33] measured cellular cross sections and derived the amounts of volume change from this data. The values for glial swelling range from 10–30% [24, 29] to as much as 40% [33]. The highest values for astrocyte swelling are measured when KCl perfusion triggers SD, while the smaller values belong to SD caused by artery occlusion. Our simulation protocol of pump interruption corresponds more to the latter, so the agreement is quite good.

Recovery of the neuronal volume is relatively fast, while the glia cell is still significantly swollen after 500 sec. This is in line with the experimental findings where nonuniform recovery times for neuronal and glial swelling were observed [24, 25]. Swelling of the whole tissue is often mentioned in the literature and known to be potentially damaging [19, 23]. Yet to our knowledge there is no SD study in which this is explicitly measured. However, our model suggests that SD could indeed lead to brain swelling.

As we have argued in Sec. “Donnan equilibrium …”, volume changes are related to anion fluxes. In Fig 8a, the changes in the Cl^−^ content in the three compartments are shown. The slow uptake of Cl^−^ by the neuron up until the recovery point is a byproduct of the transition to FES. It is also indirectly seen in Fig 5a where the Nernst potential *E*_*Cl*_ slowly depolarizes. The significantly higher uptake of Cl^−^ by the glia cell is driven by K^+^ buffering, which sets in as soon as the glia cell starts working. Swelling of both cells goes on until the repolarization point is reached (dynamics between the shaded region and the vertical pink line). A comparison of Figs 7c and 8a reveals the correlation between Cl^−^ changes and volume dynamics during SD. This is another confirmation of the link between anion fluxes and cell swelling that we pointed out in Figs 3 and 4. The glial compartment swells more than the neuron because it buffers significantly higher amounts of Cl^−^. The neuron releases K^+^ and takes up Na^+^ in large numbers. The net uptake of ions equals the uptake of Cl^−^ and is much smaller for the neuron than for the glia cell. This effect depends on the specific choice of the Cl^−^ uptake ratio and becomes weaker for smaller *χ*.

These changes in Cl^−^ concentrations go along with Cl^−^ currents. Accordingly, the recovery of cellular volumes that sets in after the repolarization point is accompanied by outward Cl^−^ currents. They are in the order of a few *μ*A/cm^2^ and currents of this magnitude are often measured in experiments. They are commonly interpreted as mechanisms of regulatory volume decrease (RVD) that are thought to be triggered by cell swelling or membrane stretching [13, 15, 17, 19, 20, 23, 70]. Our model does not assume such current triggers, but merely relies on K^+^ regulation and its byproducts. Since anion fluxes and cellular volume are intrinsically connected, literally any process that reduces cellular volumes must come with such currents. The question if they take the lead in RVD seems quite subtle and cannot be addressed in our model. It is, however, noteworthy that for depolarization–induced swelling our study does not support the hypothesis of stretch–gated or volume–sensitive Cl^−^ channels that simply open in response to cell deformation as a volume recovery mechanism. We have tested this for the isolated neuron model, i.e., the model from Fig 5 without glia that shows stable FES. Once the cell is in FES, ‘opening’ the Cl^−^ channels more by increasing *g*_*Cl*_ does not reduce swelling. What is needed is a mechanism that recovers the membrane potential first. Only then can fluxes through the Cl^−^ channels decrease the volume of the swollen cell. In other words, our model suggests that the involvement of Cl^−^ channels in RVD after SD is necessary, but not sufficient.

In Fig 8b we explore the effect of *χ* and reverse the ratio of Na^+^ release and Cl^−^ uptake. Now only 20% of the charge transported during K^+^ buffering is compensated by Cl^−^ uptake (*χ* = 0.2). Instead more Na^+^ is released. The simulation shows that this inhibits recovery from the depolarized state. The system only recovers for *χ* > 0.35, and for such values the glia cell always swells significantly more than the neuron (not shown). A value of about 0.8 seems more reasonable than 0.35 though, because experimental data suggests an abundance of anion channels in astrocytes [1, 4, 14, 35]. So a rather high value of *χ* is necessary and expected, and consequently glial swelling will always be dominant.

It is worth noticing that a similar evolution of the neuronal membrane potential as in Fig 8b is observed in recovery failure during AD in higher brain regions such as neocortex during ischemic stroke even when oxygen and glucose supply is restored. Neurons in the lower brain regions such as hypothalamus on the other hand recover after oxygen and glucose supply is restored [71, 72]. Yet there is no indication that glia takes up fewer anions in poorly recovering brain regions. Instead different morphological properties or pump isoforms may provide an explanation for different recovery behaviors [40, 46].

We remark that blocking the neuronal Cl^−^ channels does not alter the course of SD significantly. Neuronal swelling is prevented but the system undergoes the same sequence of depolarization, temporary FES, and abrupt repolarization accompanied by ECS shrinkage, which in this case, is only due to astroglial swelling (not shown). This possibility of SD without neural swelling has also been shown experimentally [69]. We conclude that the neural Cl^−^ dynamics are merely an insignificant byproduct of the cation–dominated FES–transition. In contrast, astrocytic anion uptake is essential and without it recovery may fail.

## Discussion

SD is the most extreme example of recoverable pathological ion dynamics in nervous tissue. The question of cell damage in general SD or the special case of AD depends crucially on the extent of cell swelling during these events [1, 18, 24, 25]. We have developed a simple new model that reproduces many aspects of volume dynamics during SD and is derived from known physiological facts and first physical principles. Despite its simplicity, the model’s agreement with experimental studies is impressive. In the following, we comment on some of them.

In Figs 3 and 4 we have demonstrated that cells are not expected to change volume when the Cl^−^ channels are blocked. In an SD–related study it was shown that dendritic beading depends on chloride and can be inhibited by blocking Cl^−^ cotransport [69]—without preventing SD itself. This is similar to our simulation from Fig 4 that shows depolarization without swelling. While this study is rather close to what we have simulated, Ref. [20] supports our findings by showing an inverse scenario. In this study, swelling of cortical neurons in mice is induced by a hypotonic challenge. After the osmotic stress is over, the cell shrinks again. However, when the outward rectified volume–sensitive Cl^−^ channels are blocked, the volume decrease is inhibited, which indirectly confirms our conclusion about the intrinsic connection of anion fluxes and volume dynamics. The same connection between volume regulation and Cl^−^ fluxes has been observed in astrocytes as well [16].

Numerous studies report on volume–sensitive or stretch–gated anion channels that are found not only in neurons [20] and glia [13, 15, 17], but all types of cells [70]. When cells are swollen, the subsequent RVD goes along with Cl^−^ release. It is often hypothesized that outward–rectifying volume–sensitive ion channels generate these Cl^−^ (and other anion) fluxes, and that they play an active role in volume recovery. The concept of stretch–gating assumes mechanosensitive channels that open because of membrane tension. Such questions are beyond our study, because the only active regulatory mechanisms in our model are astroglial K^+^ buffering (with slow re–release after SD) and the ion pumps. There is no explicitly volume–dependent Cl^−^ transport, but only cotransport with the buffered K^+^ (see Eq (46)). However, our model makes it clear that anion fluxes during cellular volume changes are inevitable. Their occurrence alone may be coincidental and does not directly imply an active role in volume control. We also remark that a simple stretch–gating mechanism that assumes an increased Cl^−^ conductance for a swollen cell does not recover depolarization–induced swelling (see paragraph above our discussion of Fig 8b).

While there is wide agreement that neurons swell significantly during and because of SD, there are partly contradicting experimental studies regarding the astrocytes. In Ref. [34] Takano et al. recorded hypoxia–induced SDs in living mice. Neurons swelled markedly, but the astrocytes seemed to retain their volume. This is not consistent with our model. However, there are many other experimental studies of brain slices, stroke– and ischemia–induced *in vivo* SDs, and injury models that show the opposite: astrocytes swell more and remain swollen for longer time than neurons [23–25, 33]. Our mathematical model clearly reproduces such behavior (see Fig 7). Although the model does not produce both types of volume response, we will now see how it allows us to resolve this discrepancy and provide a clear answer to the question why Takano et al. did not observe any glial swelling in their experiments.

The first thing to note is that they observed SD in the most intact tissue. In Refs. [23–25, 33] either isolated brain slices or injury and stroke models were used. This could imply that some regulation mechanisms are impaired. Takano et al. hypothesized that the astrocytes do not swell because they express a large number of volume–sensitive channels that control the volume. We know that this cannot be the right explanation though, because regardless of the specific channel properties the astrocytes must take up large amounts of K^+^ and Cl^−^ (or other anions like bicarbonate). Astrocytes cannot violate electroneutrality and consequently K^+^ buffering should at least lead to a short episode of swelling. There is no way the glia channels can accomplish efficient K^+^ buffering and prevent swelling simultaneously.

Since a large net uptake of particles is inevitable during buffering, the only way swelling can be prevented is removal of K^+^ (and Cl^−^) away from the SD site. Spatial buffering and vascular K^+^ regulation are the obvious candidates of mechanisms that provide this type of removal. In spatial buffering K^+^ is transported between glial cells from a region of high concentration to a region of low concentration. In vascular coupling, the local amount of K^+^ ions is reduced by transport along the blood vessels. Our model includes neither of these mechanisms. In particular, vascular function will be compromised in the stroke penumbra and during ischemia, and in brain slice experiments there is no functional vasculature at all. This is why our model agrees with Refs. [23–25, 33]. So even though our model does not include vasculature or spatial buffering, it implies the following new hypothesis: nonlocal mechanisms of ion regulation can prevent glia swelling in SD, while volume–sensitive ion channels cannot.

In the experiments that report on astrocyte swelling, the correlation between the SD and glial volume dynamics seems to be a question of debate. While it is commonly observed that glia cells swell long–lastingly, the neural volumes recover faster and the dynamics appear to be correlated more directly with the SD event. Hence, in Ref. [33] Zhou et al. claim that transient swelling during SD occurs only in neurons, but not in astrocytes. At first this sounds utterly contradictory to our findings from Fig 7, but a closer look reveals a more differentiated picture. In their brain slice experiments Zhou et al. induced SD by perfusion with high KCl. Slow perfusion does not trigger SD and only the astrocytes swell slowly. In contrast, fast perfusion triggers SD and the neuron shows fast correlated swelling and recovery. Glial volume changes are similar to the slow perfusion case. The astrocytic and neuronal swelling amplitudes are about 40% and 10%, respectively.

The authors conclude that there is no correlation between SD and glial swelling. We would like to suggest the following alternative interpretation of these experiments. The glia cell responds to KCl perfusion by uptake of K^+^ (and Cl^−^), which leads to swelling. Glial swelling is limited due to the finite size of the cellular membrane. The glia cell reaches its maximal size already by taking up the ions from the KCl solution. When SD occurs it leads to an additional release of K^+^ into the ECS which must be buffered. Since further glial swelling is impossible, the astrocytes build up osmotic pressure. Note that testing this scenario explicitly is beyond the scope of our model, because we have not included constraints on the cellular volumes and there is no possibility to build up higher osmotic pressure in the glial compartment. Clearly it would be an interesting future study to formally confirm our interpretation.

**Fig 9:**
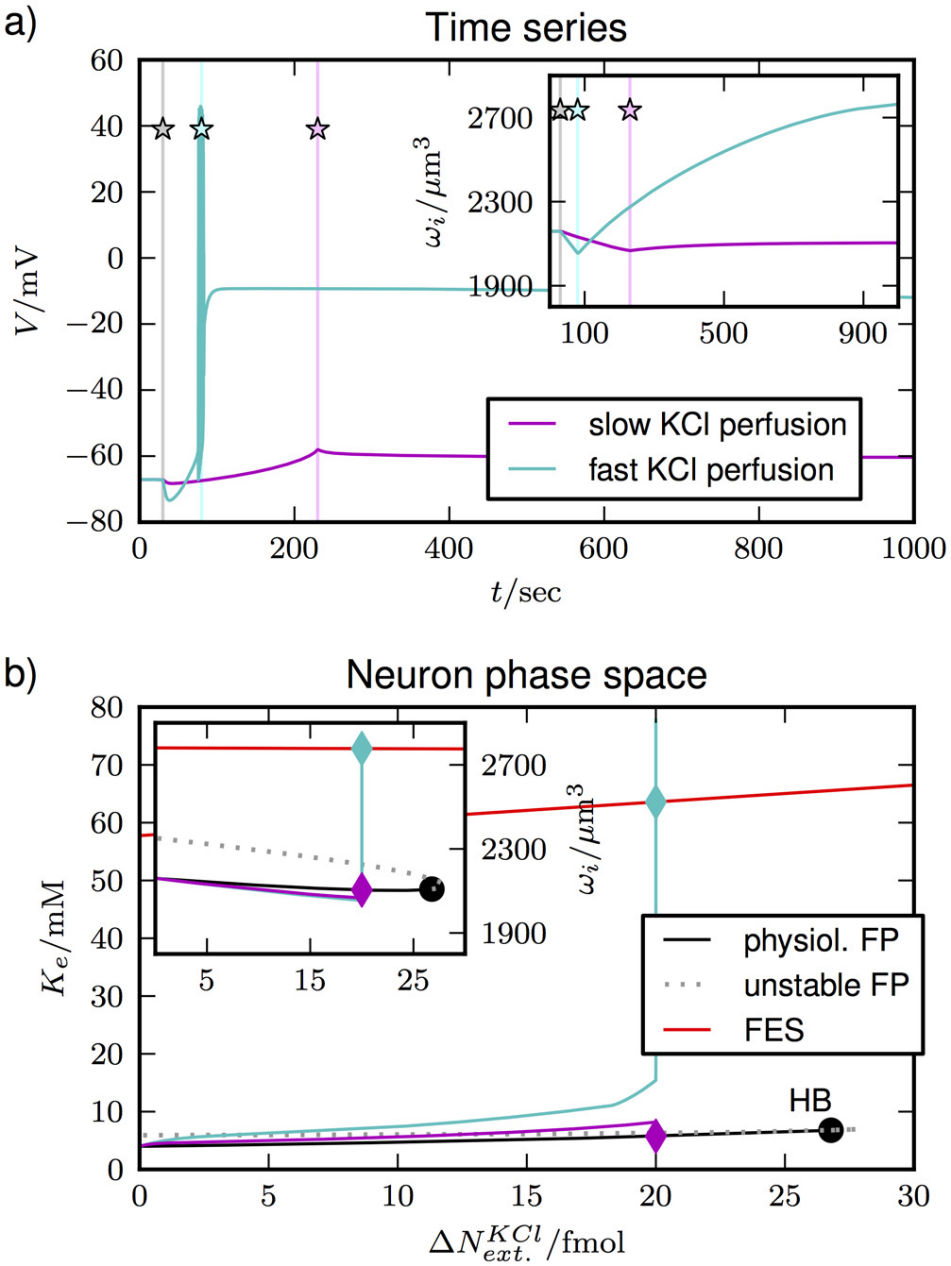
Simulation of the neuronal response to KCl perfusion. For the slow perfusion case in **(a)** 20 fmol of KCl are added within 200 sec (pink curve). The beginning and end of KCl addition are marked by the vertical lines (with stars). The neuron retains its polarization and volume (inset). For fast perfusion (turquoise curve) the same amount of KCl is added between 20 and 70 sec (vertical lines marked with stars). This induces depolarization and swelling. In **(b)** this is related to the fixed point structure. The black and red section of the fixed point curve indicate stable physiological conditions and FES, respectively. With slow perfusion the neuron remains on the physiological branch, with fast perfusion it goes into FES. *K*_*e*_ and *ω*_*i*_ (inset) are significantly increased.

The behavior of the neuron can be understood separately. In Fig 9, we have simulated the slow and fast perfusion protocol by adding 20 fmol of KCl within 200 sec and within 50 sec. This external KCl addition is measured by $\Delta N_{\text{ext}.}^{\text{KCl}}$:

$$\begin{array}{ccc} N_{e}^{\text{K}} & = & N_{e}^{\text{K,0}}+N_{i}^{\text{K,0}}-N_{i}^{\text{K}}+\Delta N_{\text{ext}.}^{\text{KCl}}\;, \end{array}$$ (55)

$$\begin{array}{ccc} N_{e}^{\text{Cl}} & = & N_{e}^{\text{Cl,0}}+N_{i}^{\text{Cl,0}}-N_{i}^{\text{Cl}}+\Delta N_{\text{ext}.}^{\text{KCl}}\;. \end{array}$$ (56)

The glial compartment is not included in this simulation, which means that we only consider the SD ignition process: the transition to FES.

The time series are shown in Fig 9a. For the pink curve, KCl is slowly added between 30 and 230 sec (vertical grey and shaded pink line with star, respectively). We see that the neuron retains its polarization. For the turquoise curve, we have applied fast perfusion between 30 and 80 sec (vertical turquoise line with star). The cell depolarizes and goes into FES. Only in the latter case does the neuron swell significantly (inset). This is the exact same behavior that Zhou et al. observed. The explanation of these different behaviors is a combination of our new understanding of cellular volume dynamics and previous theoretical results on neuronal responses to K^+^ elevation [43].

In Fig 9b, we show the fixed point structure of the system and the time series from Fig 9a in relation to it. We have already pointed out in Fig 5b that the model without the glia has two stable fixed point branches corresponding to a stable physiological state and FES. In Fig 9b, we find a similar structure for the variation of $\Delta N_{\text{ext}.}^{\text{KCl}}$. The physiological state can theoretically be stable for a KCl elevation $\Delta N_{\text{ext}.}^{\text{KCl}}$ of up to more than 25 fmol. Only for higher values of $\Delta N_{\text{ext}.}^{\text{KCl}}$ the physiological state becomes unstable in a Hopf bifurcation (HB). For slow perfusion (pink trajectory), the trajectory of the neuron is guided by the physiological fixed point branch. The neuron maintains a low level of *K*_*e*_ by taking up most of the K^+^ into the cell. This goes along with the release of Na^+^ such that the net flux of particles is almost zero (not shown). That is why the volume remains almost constant (see insets in Fig 9a and 9b).

When K^+^ is added rapidly, the neuron cannot make up for the elevation any longer. The trajectory deviates from the physiological branch and *K*_*e*_ passes the threshold for the transition to FES (turquoise trajectory). Only then the neuron depolarizes and Cl^−^ flows into the cell. This results in neuronal swelling (see inset).

To conclude, by combining an established semi–phenomenological neuron–glia description and first physical principles in a consistent way, we have developed a remarkably simple, yet physiologically relevant model for neural and glial volume dynamics. Despite its simplicity this new model describes many aspects of the interplay between the neuron, astrocytes, and anion channels during SD accurately. There is strong experimental evidence in support of our study, and we emphasize that most of our explanations come from general physical principles (osmosis, electroneutrality) rather than biophysical details (volume–sensitive anion channels, K^+^/Cl^−^–cotransport). Accordingly, we claim that our theory is broadly applicable and may be used as a future guide to interpret swelling processes in brain pathologies.
